# Six *Pseudoalteromonas* Strains Isolated from Surface Waters of Kabeltonne, Offshore Helgoland, North Sea

**DOI:** 10.1128/genomeA.01697-15

**Published:** 2016-02-11

**Authors:** Melissa B. Duhaime, Antje Wichels, Matthew B. Sullivan

**Affiliations:** a Department of Ecology and Evolutionary Biology, University of Michigan, Ann Arbor, Michigan, USA; b Biologische Anstalt Helgoland, Alfred Wegener Institute, Helgoland, Germany; c Department of Microbiology, The Ohio State University, Columbus, Ohio, USA; d Department of Civil, Environmental and Geodetic Engineering, The Ohio State University, Columbus, Ohio, USA

## Abstract

Draft genomes are presented for 6 *Pseudoalteromonas* sp. strains isolated from surface waters at Kabeltonne, Helgoland, a long-term ecological research station in the North Sea. These strains contribute knowledge of the genomic underpinnings of a developing model system to study phage-host dynamics of a particle-associated ocean copiotroph.

## GENOME ANNOUNCEMENT

Members of the genus *Pseudoalteromonas* (*Gammaproteobacteria*; *Alteromonadales*; *Alteromonadaceae*) are Gram-negative aerobic chemoorganoheterotrophs of marine microbial communities (1). They span habitats ranging from surface (2) to deep sea (3), polar waters (4) and sea ice (5), and temperate salterns (6). While comprising up to 6% of free-living seawater communities in polar regions (2), *Pseudoalteromonas* spp. are specialized in surface-associated habitats (7). *Pseudoalteromonas* spp. are prolific biofilm formers and are found among the dominant community members living on ocean particles (8) and on surfaces of marine eukaryotes (9, 10). They produce myriad bioactive compounds (11) that can prohibit settlement of fouling larvae and algae (9), presumably to give them a competitive advantage in biofilms. Their ability to utilize diverse and complex carbon substrates (12) likely enables their capacity to boom as r-strategist copiotrophs when potential carbon sources become available (13), for example, in the wake of the Deepwater Horizon oil spill (14), in the oil-polluted North Sea (15), or in decaying phytoplankton blooms (9). There are 49 established species in the genus, though 3,174 strains have not been described at the species level (January 2015, GenBank). Of these, 15 and 28 genomes have been sequenced, respectively (January 2015, GenBank). This *Pseudoalteromonas* sequencing project is a step in the development of a model system to study the ecology and evolution of these particle-associated ocean copiotrophs and their viruses (16, 17, M. B. Duhaime et al., submitted for publication).

DNA was extracted from these nonpigmented clonal isolates using the Qiagen DNeasy blood and tissue kit (Qiagen, USA). Indexed paired-end sequencing libraries were prepared with an Illumina TruSeq DNA sample preparation kit (Illumina, Inc., USA) and sequenced on an Illumina HiSeq2000 platform. The 100-bp reads were quality trimmed using sickle version 1.33 (18) with no 5′ trimming, and all sequences with Ns or <Q20 were removed. The remaining singleton and paired reads were assembled using Velvet version 1/1/07 (19). Contigs from the velvet assemblies were used to generate *in silico* jumping libraries (1,000 kb) to be further assembled with the original reads using ALLPATHS-LG (20). Proteins were predicted and annotated using NCBI’s PGAP pipeline (December 2015; http://www.ncbi.nlm.nih.gov/books/NBK174280). Genome completeness was estimated with CheckM (21).

To further develop the Helgoland *Pseudoalteromonas* phage-host system, prophages were predicted with VirSorter and the RefSeq virus database (22). One prophage was predicted in strains 13-15 and H103 (15.7 and 55 kb, respectively) and two in strain H105 (87.3 and 15.6 kb). Functional annotations were screened for insights into potential substrates (12). All strains contain at least one outer membrane phospholipase and endoglucanase, which are involved, respectively, in hemolysis/bacteriocin release (23) and hydrolysis of cellulose, a major plant cell wall component (24). A glyoxalase of the beta-lactamase superfamily involved in antibiotic resistance is also found in all strains.

### Nucleotide sequence accession numbers.

This whole-genome shotgun project has been deposited in GenBank under the following accession numbers: LODJ00000000 (*Pseudoalteromonas* sp. strain 13-15), LODI00000000 (strain H71), LODK00000000 (strain H100), LOFG00000000 (strain H103), LOFH00000000 (strain H105), and LOFI00000000 (strain 10-33). This paper describes the first versions.
